# Hesperidin Exhibits Protective Effects against PM_2.5_-Mediated Mitochondrial Damage, Cell Cycle Arrest, and Cellular Senescence in Human HaCaT Keratinocytes

**DOI:** 10.3390/molecules27154800

**Published:** 2022-07-27

**Authors:** Herath Mudiyanselage Udari Lakmini Herath, Mei Jing Piao, Kyoung Ah Kang, Ao Xuan Zhen, Pincha Devage Sameera Madushan Fernando, Hee Kyoung Kang, Joo Mi Yi, Jin Won Hyun

**Affiliations:** 1 Department of Biochemistry, College of Medicine, Jeju National University, Jeju 63243, Korea; lakmini@stu.jejunu.ac.kr (H.M.U.L.H.); mjpiao@jejunu.ac.kr (M.J.P.); legna07@jejunu.ac.kr (K.A.K.); zhenaoxuan705@stu.jejunu.ac.kr (A.X.Z.); sameera@stu.jejunu.ac.kr (P.D.S.M.F.); 2 Jeju Research Center for Natural Medicine, Jeju National University, Jeju 63243, Korea; pharmkhk@jejunu.ac.kr; 3 Department of Pharmacology, College of Medicine, Jeju National University, Jeju 63243, Korea; 4 Department of Microbiology and Immunology, Inje University College of Medicine, Busan 47392, Korea; jmyi76@inje.ac.kr

**Keywords:** hesperidin, PM_2.5_, cell cycle arrest, senescence

## Abstract

Particulate matter 2.5 (PM_2.5_) exposure can trigger adverse health outcomes in the human skin, such as skin aging, wrinkles, pigment spots, and atopic dermatitis. PM_2.5_ is associated with mitochondrial damage and the generation of reactive oxygen species (ROS). Hesperidin is a bioflavonoid that exhibits antioxidant and anti-inflammatory properties. This study aimed to determine the mechanism underlying the protective effect of hesperidin on human HaCaT keratinocytes against PM_2.5_-induced mitochondrial damage, cell cycle arrest, and cellular senescence. Human HaCaT keratinocytes were pre-treated with hesperidin and then treated with PM_2.5_. Hesperidin attenuated PM_2.5_-induced mitochondrial and DNA damage, G_0_/G_1_ cell cycle arrest, and SA-βGal activity, the protein levels of cell cycle regulators, and matrix metalloproteinases (MMPs). Moreover, treatment with a specific c-Jun N-terminal kinase (JNK) inhibitor, SP600125, along with hesperidin markedly restored PM_2.5_-induced cell cycle arrest and cellular senescence. In addition, hesperidin significantly reduced the activation of MMPs, including MMP-1, MMP-2, and MMP-9, by inhibiting the activation of activator protein 1. In conclusion, hesperidin ameliorates PM_2.5_-induced mitochondrial damage, cell cycle arrest, and cellular senescence in human HaCaT keratinocytes via the ROS/JNK pathway.

## 1. Introduction

Air pollutants such as particulate matter (PM), nitrogen oxide, carbon monoxide, volatile organic compounds, ground-level ozone, and cigarette smoke are serious public health problems. PM readily penetrates human body barriers and causes harmful effects on the skin [1]. Inhaled fine particulate matter 2.5 (PM_2.5_; aerodynamic diameter <2.5 μm) can penetrate deep lung tissues and may even enter the bloodstream. As a result, PM_2.5_ damages the respiratory, nervous, cardiovascular, and immune systems [2,3,4,5]. Additionally, PM_2.5_ causes skin disorders, including atopic dermatitis, acne, and psoriasis [6]. PM_2.5_ is a complex combination of chemical substances, including metals, polycyclic aromatic hydrocarbons (PAHs), allergens, and endotoxins [7]. PAHs in PM_2.5_ directly penetrate the skin barrier via aryl hydrocarbon receptors (AhRs) and increase reactive oxygen species (ROS) production [7]. Excessive ROS production causes DNA damage, lipid peroxidation, skin senescence, and inflammation [8].

A previous in vitro study revealed that PM_2.5_ inhibits cell growth by decreasing cell proliferation and/or triggering cell death [9]. The reduction in cell proliferation can occur at various steps during the cell cycle. Cell cycle progression is determined at different phases by “cell cycle checkpoints”, which evaluate whether the previous phase was successfully completed and decide whether the cell should progress to the next phase of the cell cycle or be blocked [10]. Genotoxic stressors and structural protein dysfunctions can delay or inhibit the cell cycle. ROS-induced oxidative DNA damage induces cell cycle arrest at the G_0_/G_1_ phase in human HaCaT keratinocytes [11,12]. Additionally, stable cell cycle arrest leads to cellular senescence and contributes to aging [13]. However, limited studies have been conducted to demonstrate the effect of PM_2.5_ on the inhibition of cell proliferation via cell cycle arrest in human HaCaT keratinocytes.

Hesperidin (3, 5, 7-trihydroxyflavone 7-rhamnoglucoside) is the main polyphenol compound found in citrus fruits. This bioflavonoid exhibits antioxidant, anti-inflammatory, and antibacterial effects [14,15]. A recent study has demonstrated that hesperidin protected cells against redox imbalances [16]. In addition, hesperidin markedly reduces oxidative stress-induced inflammatory cytokine levels and apoptosis [17,18]. This study aimed to investigate the protective effects of hesperidin against PM_2.5_-induced cell cycle arrest and cellular senescence in HaCaT keratinocytes.

## 2. Results

### 2.1. Hesperidin Alleviates PM_2.5_-Induced Mitochondrial Damage

**Figure 1:**
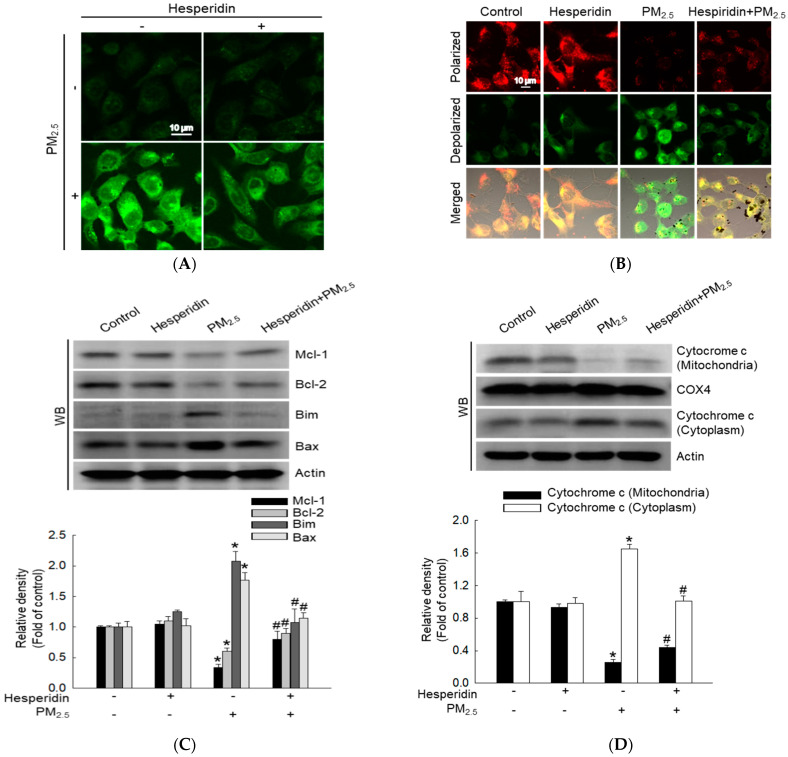
Effect of hesperidin on PM_2.5_-induced mitochondrial damage. Cells were pre-treated with hesperidin (50 µM) for 30 min and then with PM_2.5_ (50 μg/mL) for 24 h and analyzed using confocal microscopy to assess (**A**) mitochondrial ROS (DHR123 staining) and (**B**) mitochondrial membrane potential (Δψ_m_) (JC-1 staining). Western blotting (WB) indicates the expression of (**C**) myeloid cell leukemia-1 (Mcl-l), B-cell lymphoma 2 (Bcl-2), Bcl-2-like protein 11 (Bim), Bcl-2-associated X protein (Bax) proteins, and (**D**) cytochrome c protein in mitochondrial and cytosolic fractions. Cytochrome c oxidase IV (COX4) and actin were used as loading controls. * *p* < 0.05 compared with the control group and ^#^ *p* < 0.05 compared with the PM_2.5_-treated group.

A previous study reported that hesperidin (50 µM) exhibited a cytoprotective effect in human HaCaT keratinocytes against ultraviolet B (UVB)-induced oxidative stress and did not induce any cytotoxicity [18]. Therefore, we selected 50 µM hesperidin as the optimum concentration in this study. Cells were pre-treated with hesperidin (50 µM) for 30 min and then with PM_2.5_ (50 μg/mL) for 24 h, and the cells were analyzed using confocal microscopy to assess the effect of hesperidin on PM_2.5_-induced mitochondrial damage. Confocal microscopy images indicated that hesperidin significantly reduced PM_2.5_-induced mitochondrial ROS generation (Figure 1A). The mitochondrial membrane potential (Δψ_m_) was measured using JC-1 staining. Red fluorescence indicates mitochondrial polarization and green fluorescence indicates mitochondrial depolarization [19,20]. Hesperidin notably reversed PM_2.5_-induced mitochondrial depolarization (Figure 1B). Furthermore, hesperidin markedly restored PM_2.5_-reduced myeloid cell leukemia-1 (Mcl-1), and B-cell lymphoma 2 (Bcl-2), and hesperidin markedly inhibited the expression of Bcl-2-like protein 11 (Bim) and Bcl-2-associated X protein (Bax) induced by PM_2.5_ (Figure 1C). In addition, PM_2.5_ increased the release of cytochrome c from the mitochondria to the cytoplasm; however, hesperidin attenuated it (Figure 1D). These results confirm that hesperidin remarkably alleviates PM_2.5_-induced mitochondrial damage.

### 2.2. Hesperidin Reduces PM_2.5_-Induced G_0_/G_1_ Cell Cycle Arrest

**Figure 2:**
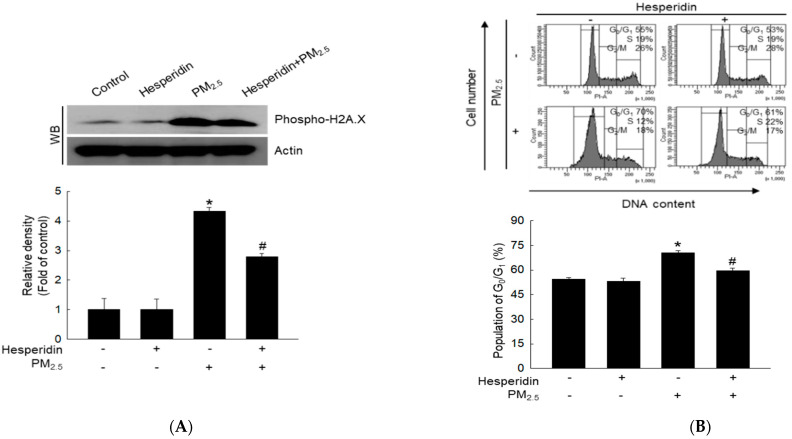

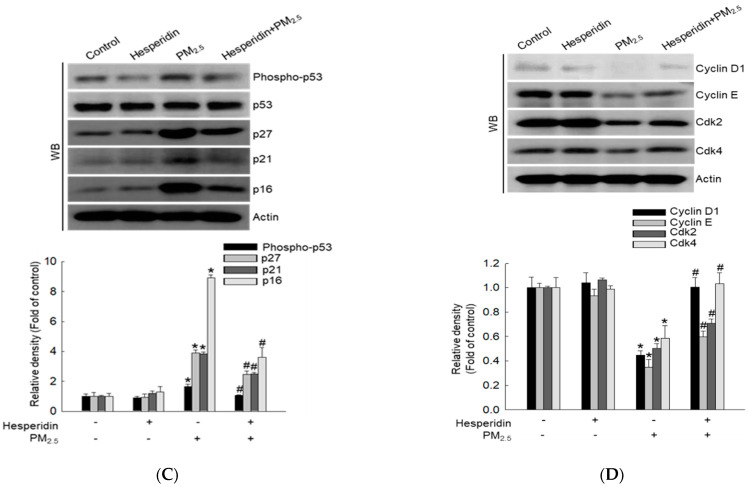
Effect of hesperidin on PM_2.5_-induced G_0_/G_1_ cell cycle arrest. Cells were pre-treated with hesperidin (50 µM) for 30 min and then with PM_2.5_ (50 μg/mL) for 48 h and (**A**) cell lysates were analyzed to detect phosphor-H2A.X using western blot (WB). (**B**) Cellular DNA was stained with propidium iodide (PI) solution and analyzed using flow cytometry. Cell lysates were analyzed for (**C**) phosphor-p53, p53, p27, p21, and p16, and (**D**) cyclin D1, cyclin E, Cdk2, and Cdk4 proteins were detected using western blot (WB). Actin was used as a loading control. * *p* < 0.05 compared with the control group and ^#^ *p* < 0.05 compared with the PM_2.5_-treated group.

PM_2.5_ induces mitochondrial dysfunction, resulting in cellular oxidative stress and cellular energy supply inhibition [21]. In addition, oxidative stress causes DNA damage and arrests the cell cycle in the G_0_/G_1_ phase [22,23]. Phospho-H2A.X is a biomarker of DNA double-strand breaks [24]. Therefore, we evaluated the effect of hesperidin on PM_2.5_-induced DNA damage by measuring the expression of phospho-H2A.X protein using western blotting. Western blot results indicated that PM_2.5_ promoted phospho-H2A.X expression, whereas hesperidin reversed this effect (Figure 2A).

Additionally, DNA damage is closely associated with cell cycle alterations in mammalian cells [25,26]. Hence, we evaluated the effects of PM_2.5_ on cell cycle alterations in HaCaT keratinocytes using flow cytometry. The results indicated that after PM_2.5_ treatment, 70% of the cells were arrested in the G_0_/G_1_ phase of the cell cycle compared with that of the control group, and hesperidin decreased this arrest to 61% (Figure 2B). Furthermore, at the molecular level, PM_2.5_ increased the expression of phospho-p53, p27, p21, and p16 proteins (Figure 2C) but decreased the expression of other cell cycle regulatory proteins, including cyclin D1, cyclin E, cyclin-dependent kinase 2 (Cdk2), and cyclin-dependent kinase 4 (Cdk4) (Figure 2D). Hesperidin decreased PM_2.5_-induced increase in phospho-p53, p27, p21, and p16 protein levels and restored the reduced cyclin D1, cyclin E, Cdk2, and Cdk4 protein levels. These results indicated that hesperidin alleviates PM_2.5_-induced cell cycle arrest.

### 2.3. Hesperidin Alleviates PM_2.5_-Induced Cellular Senescence

**Figure 3:**
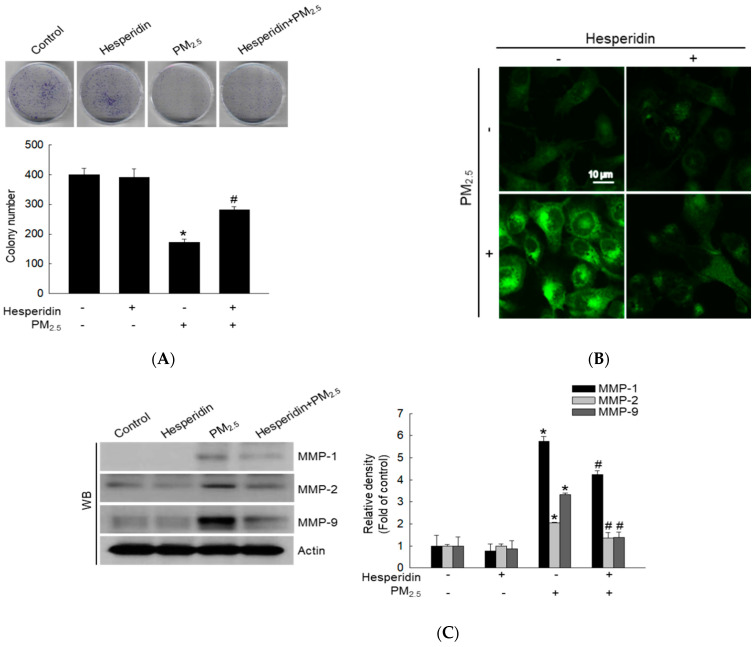
Effect of hesperidin on PM_2.5_-induce cellular senescence. Cells were pre-treated with hesperidin (50 µM) and then with PM_2.5_ (50 μg/mL). (**A**) Cells were treated with hesperidin and PM_2.5_ for 7 days, and colonies were stained with Diff-Quik staining. (**B**) Cells were stained for β-galactosidase activity using SPiDER-βGal and analyzed using confocal microscopy. (**C**) Western blotting (WB) was performed to detect matrix metalloproteinases (MMP)-1, MMP-2, and MMP-9 proteins. Actin was used as a loading control. * *p* < 0.05 compared with the control group and ^#^ *p* < 0.05 compared with the PM_2.5_-treated group.

Mitochondrial dysfunction is associated with cellular senescence and oxidative stress-mediated cell cycle arrest [27]. PM_2.5_ was reported to induce G_0_/G_1_ cell cycle arrest, mitochondrial damage, and cellular senescence [28]. Oxidative stress induced by PM_2.5_ significantly reduced colony formation, with a reduced number and size of keratinocyte cell colonies compared with that of the control group (Figure 3A). As depicted in Figure 3B, the PM_2.5_-treated group exhibited higher senescence-associated β-galactosidase (SA-βGal) activity (increased green fluorescence) levels whereas the hesperidin-treated group exhibited reduced SA-βGal activity levels. Additionally, matrix metalloproteinase (MMP) production accelerates cellular senescence [29]; therefore, we evaluated the expression levels of MMP-1, -2, and -9 using western blotting. MMP-1, -2, and -9 protein levels were significantly enhanced by PM_2.5_, and this effect was reversed by hesperidin (Figure 3C).

### 2.4. Hesperidin Decreases PM_2.5_-Induced JNK Signal Transduction

**Figure 4:**
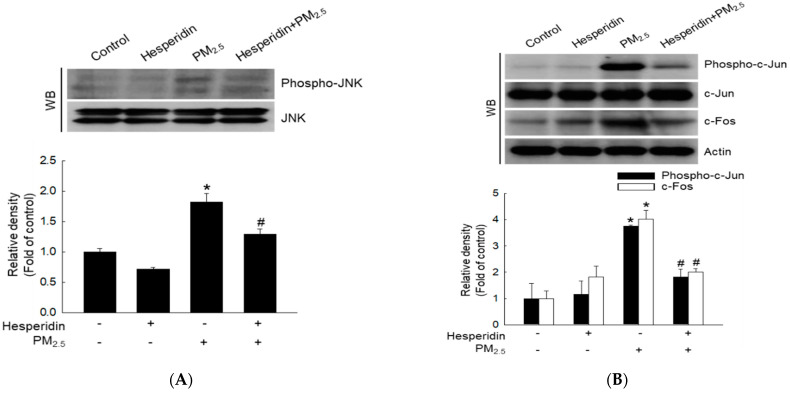
Effect of hesperidin on PM_2.5_-induced JNK signal transduction. Cells were pre-treated with hesperidin (50 µM) for 30 min and then with PM_2.5_ (50 μg/mL) for 48 h, and cell lysates were analyzed using western blot (WB) for (**A**) phosphor-JNK and JNK and (**B**) phosphor-c-Jun, c-Jun, and c-Fos. Actin was used as a loading control. * *p* < 0.05 compared with the control group and ^#^ *p* < 0.05 compared with the PM_2.5_-treated group.

The c-Jun N-terminal kinase (JNK) signaling pathway is a subcategory of the mitogen-activated protein kinase (MAPK) pathway, which is important for cell cycle progression and senescence. We quantified proteins involved in the JNK pathway using western blotting to determine the effect of hesperidin on PM_2.5_-induced JNK signal transduction. PM_2.5_ stimulation increased phosphorylated JNK protein levels (Figure 4A). Additionally, we evaluated the activity of the downstream activator protein 1 (AP-1) transcription factor by evaluating the expression of c-Jun and c-Fos. PM_2.5_ promoted the expression of phospho-c-Jun and c-Fos proteins (Figure 4B). In contrast, hesperidin reduced JNK signal transduction, by decreasing phospho-c-Jun, and c-Fos protein levels (Figure 4A,B).

**Figure 5:**
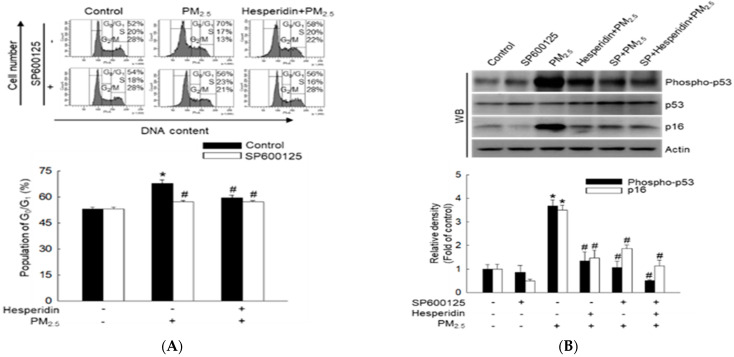

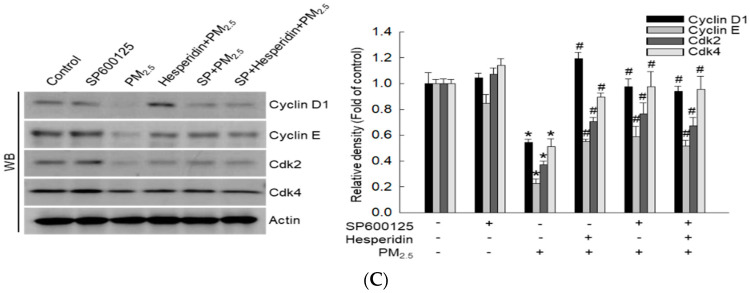
Effect of specific inhibitor (SP600125) of JNK on PM_2.5_-induced cell cycle arrest in G_0_/G_1_. Cells were pre-treated with hesperidin (50 µM) or with SP600125 (5 µM) for 30 min and then with PM_2.5_ (50 μg/mL) for 48 h. (**A**) Cellular DNA was stained with propidium iodide (PI) and analyzed using flow cytometry. Cell lysates were analyzed using western blot (WB) for (**B**) phosphor-p53, p53, p16, (**C**) cyclin D1, cyclin E, Cdk2, and Cdk4 proteins. Actin was used as a loading control. * *p* < 0.05 compared with the control group and ^#^ *p* < 0.05 compared with the PM_2.5_−treated group.

We then investigated whether PM_2.5_ caused cell cycle arrest by inducing oxidative stress and JNK signaling activation by treating the cells with the JNK pathway inhibitor, SP600125. Flow cytometric analysis indicated that both SP600125 and hesperidin significantly reduced PM_2.5_-induced cell cycle arrest by suppressing JNK (Figure 5A). Hesperidin, SP600125, and the combination of both reduced the percentage of cells arrested in the G_0_/G_1_ phase compared with that observed in the PM_2.5_-treated group. Notably, western blot results indicated that treatment with SP600125 and hesperidin reduced hosphor-p53 and p16 protein levels (Figure 5B) and increased cyclin D1, cyclin E, Cdk2, and Cdk4 protein levels (Figure 5C). Collectively, these results confirm that hesperidin reduces PM_2.5_-induced cell cycle arrest at the G_0_/G_1_ phase by inhibiting the JNK signaling pathway.

**Figure 6:**
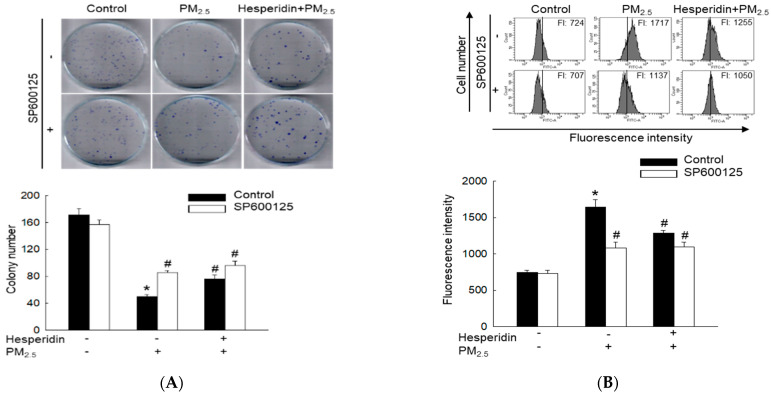
Effect of specific inhibitor (SP600125) of c-Jun N-terminal kinase (JNK) on PM_2.5_-induced human keratinocyte senescence. Cells were pre-treated with hesperidin (50 µM) or with SP600125 (5 µM) and then with PM_2.5_ (50 μg/mL). (**A**) Colonies were counted after Diff-Quik staining. (**B**) β-Galactosidase activity was assessed using flow cytometry. * *p* < 0.05 compared with the control group and ^#^ *p* < 0.05 compared with the PM_2.5_-treated group.

Cell cycle arrest contributes to the induction of cellular senescence. We confirmed that PM_2.5_-induced HaCaT cell cycle arrest occurs at the G_0_/G_1_ phase via the JNK signaling pathway. Therefore, we examined the effect of hesperidin on PM_2.5_-induced cell proliferation and senescence after inhibiting the JNK signaling pathway. HaCaT cells that were pre-treated with SP600125, hesperidin, or both exhibited enhanced colony formation with an increased number and size of HaCaT cell colonies compared with that of cells treated only with PM_2.5_ (Figure 6A). Flow cytometric analysis revealed that SP600125, hesperidin, and the combination group exhibited a significant reduction in SA-βGal activity compared with that of PM_2.5_-treated cells (Figure 6B). These results confirm that hesperidin ameliorates PM_2.5_-induced cellular senescence, as a JNK pathway inhibitor.

## 3. Discussion

The human skin is the first barrier to environmental stress. However, PM can cross this barrier and cause skin disorders. PM_2.5_ exposure increases ROS production in skin keratinocytes [30]. Excessive ROS in skin cells contributes to various biological changes, such as skin senescence and aging, through various cellular signaling cascades [31]. Additionally, prolonged cell cycle arrest leads to cellular senescence and aging [32]. Recent studies have demonstrated that hesperidin ameliorates excessive ROS levels in skin cells [18,33]. In this study, we investigated the ameliorative effects of hesperidin on PM_2.5_-induced cell cycle arrest and senescence in HaCaT keratinocytes.

Excessive ROS levels can induce oxidative stress, which causes depolarization of the mitochondrial membrane potential and negatively impacts oxidative phosphorylation. Damaged mitochondria lead to an increase in ROS levels by activating the internal mitochondrial network [34]. Moreover, hesperidin exhibits antioxidative and anti-inflammatory properties [18,33]. In our study, hesperidin effectively reduced PM_2.5_-induced mitochondrial ROS levels and restored the damaged mitochondrial membrane potential. H2A.X phosphorylation usually occurs in DNA double-strand breaks; therefore, H2A.X phosphorylation is used as a direct indicator of DNA damage [35]. Several studies have reported that PM_2.5_-induced oxidative stress causes DNA damage in HaCaT keratinocytes [8,36]. In our study, hesperidin effectively reduced PM_2.5_-induced DNA damage. Moreover, previous in vitro studies have indicated that oxidative stress contributes to mitochondrial and DNA damage, leading to cell cycle arrest in skin cells [37,38]. Similarly, we observed an increase in the number of cells in the G_0_/G_1_ phase after PM_2.5_ treatment. Additionally, hesperidin reduced the cell population in G_0_/G_1_ phase, which was increased by PM_2.5_ treatment. Hence, we analyzed G_0_/G_1_ checkpoints such as cyclin D1-Cdk4, which is necessary for progression to the G_1_ phase, and cyclin E-Cdk2, which is necessary to regulate the G_1_/S transition. Hesperidin treatment restored the expression of cyclin D1, Cdk4, cyclin E, and Cdk2. Furthermore, p27, p21, and p16 are Cdk inhibitors that promote cell cycle arrest and senescence [39]. In our study, p27, p21, and p16 were upregulated by PM_2.5_ and downregulated by hesperidin pre-treatment. These results suggest that hesperidin inhibits PM_2.5_-induced G_0_/G_1_ phase arrest.

Cellular senescence is commonly defined as an irreversible cell cycle arrest accompanied by epigenetic modifications and changes in cell morphology and secretory profile, among other things. Furthermore, cell aging refers to the gradual reduction in cellular functions and the increasing possibility of cell death overtime [40]. Permanent cell cycle arrest triggers cellular senescence as a survival response. Various stimuli trigger cellular senescence, such as oxidative DNA damage and telomere shortening [39]. Senescence is characterized by several hallmarks such as inability to proliferate, change in cell shape (flattened shape), enlarged size, vacuolization, cyclin D1 accumulation, resistance to apoptosis, senescence-associated secretory phenotype, and increased SA-βGal activity [41]. Moreover, growing evidence suggests that senescent cell accumulation causes skin aging. Matrix metalloproteinases (MMPs) are observed in chronologically aged skin and cause extracellular matrix (ECM) breakdown [42]. The MMP-1 initiates collagen fragmentation in human skin cells [43]; MMP-9 directly degrades ECM proteins and activates chemokines and cytokines [44]. Furthermore, PM_2.5_-induced oxidative stress enhances keratinocyte senescence and aging [45]. Consistent with this, in our study, PM_2.5_ increased MMP-1 and MMP-9 levels whereas hesperidin reduced them, indicating that hesperidin exhibits an inhibitory effect on PM_2.5_ -induced skin senescence and aging.

ROS can activate the MAPK signaling pathway. JNK is a member of the MAPK family and is reportedly activated by oxidative stress. JNK signaling regulates a variety of cellular processes, including cell cycle progression, proliferation, survival, senescence, and apoptosis [46]; JNK triggers p53 phosphorylation and activates p53-mediated cell cycle regulatory pathways. JNK phosphorylation induces the activation of downstream transcription factor proteins, including AP-1 protein [45]. AP-1 activation is critical for MMP synthesis [47]. In our study, we investigated AP-1 activation by evaluating the activation of c-Jun and c-Fos proteins, which are two main components of the AP-1 complex. We found that PM_2.5_ increased c-Jun and c-Fos protein levels whereas hesperidin reduced them, suggesting that hesperidin reversed PM_2.5-_induced oxidative effects. Additionally, the JNK inhibitor SP600125 effectively inhibited PM_2.5_-induced cell cycle arrest, senescence, and aging in HaCaT keratinocytes. Therefore, our results suggest that PM_2.5_-induced cell cycle arrest is mediated by the JNK pathway. Previous investigations on hesperidin’s therapeutic potential against environmental pollutants (UVA and UVB) indicated that it protects HaCaT cells by reducing intracellular ROS production, inflammatory responses, and cell apoptosis [18,33]. In the present study, we evaluated the therapeutic potential of hesperidin against PM_2.5_ and found that hesperidin is a potential therapeutic agent against both UV radiation and PM_2.5_. We believe that further investigation of the potential of hesperidin against a wide range of environmental pollutants holds significance.

**Figure 7:**
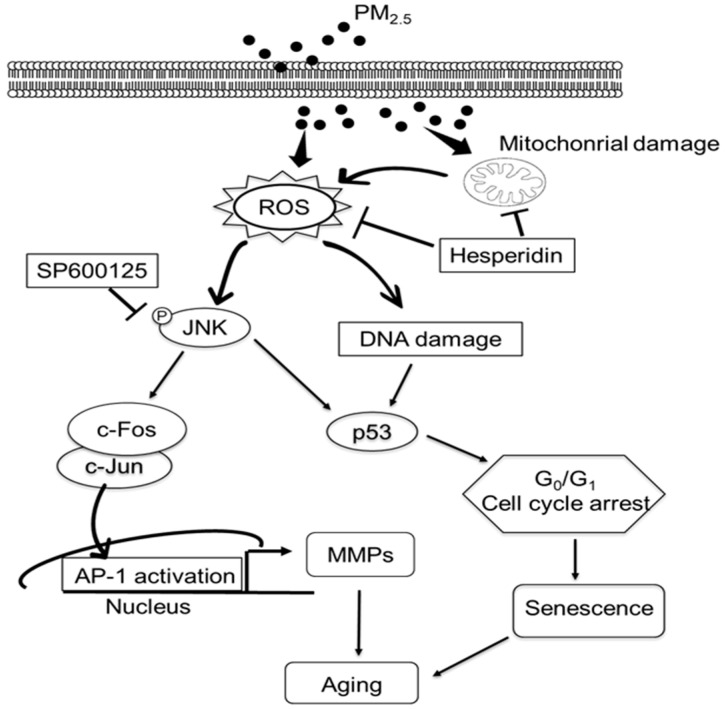
Graphical illustration of the protective effects of hesperidin on PM_2.5_-induced skin cell damage. Hesperidin ameliorates PM_2.5_-induced mitochondrial damage and reduces the intracellular ROS level. Hesperidin inhibits the PM_2.5_-induced cell cycle arrest at the G_0_/G_1_ phase and ameliorates cellular senescence.

In conclusion, our study revealed that hesperidin ameliorates PM_2.5_-induced cell cycle arrest and senescence in HaCaT keratinocytes. As depicted in Figure 7, hesperidin mitigated PM_2.5_-induced mitochondrial damage and reduced total ROS levels. Moreover, hesperidin alleviates PM_2.5_-induced DNA damage, cell cycle arrest, and cellular senescence through the ROS/JNK pathway.

## 4. Materials and Methods

### 4.1. Reagents and Antibodies

Hesperidin (C_28_H_34_O_15_) and standard diesel PM_2.5_ (SRM 1650b) were purchased from Sigma-Aldrich, Inc. (St. Louis, MO, USA) and dissolved in dimethyl sulfoxide (DMSO). PM_2.5_ was issued by the National Institute of Standards and Technology (NIST, Gaithersburg, MD, USA), and the stock solution (25 mg/mL) was sonicated for 30 min to avoid agglomeration.

### 4.2. Cell Culture

The HaCaT cell line was purchased from Cell Line Service (Heidelberg, Germany). Cells were grown at 37 °C in a humidified incubator in an atmosphere of 5% CO_2_. Cells were cultured in Dulbecco’s modified Eagle’s medium (DMEM; Gibco, Life Technologies, Grand Island, NY, USA) supplemented with 10% fetal bovine serum (FBS) and antibiotic-antimycotic (Gibco, Waltham, MA, USA).

### 4.3. Mitochondrial ROS Measurement

HaCaT cells were seeded (1.5 × 10^5^ cells/mL) in chamber slides and incubated at 37 °C for 16 h. After treatment with hesperidin (50 µM) for 30 min, cells were treated with PM_2.5_ (50 μg/mL) for 24 h. Mitochondrial ROS was detected after 30 min of staining with dihydrorhodamine 123 (DHR 123; Molecular Probes, Eugene, OR, USA). Cells were mounted on microscope slides using a mounting medium. Images were captured using a confocal microscope (Carl Zeiss; Oberkochen, Germany).

### 4.4. Mitochondrial Membrane Potential (Δψ_m_) Measurement

Cells were seeded at a density of 1.5 × 10^5^ cells/mL in chamber slides, incubated at 37 °C for 16 h, treated with hesperidin (50 µM) for 30 min, and subsequently treated with PM_2.5_ (50 μg/mL) for 24 h. The Δψ_m_ was analyzed using confocal microscopy after staining with 5,5′,6,6′ tetrachloro-1,1′,3,3′-tetraethylbenzimidazolylcarbocyanine iodide (JC-1, Invitrogen, Waltham, MA, USA), a lipophilic cationic fluorescence dye.

### 4.5. Western Blotting

After each treatment, harvested cells were subjected to total protein analysis according to a previously described method [48], then electrophoresed on 12% sodium dodecyl sulfate-polyacrylamide gels. The separated proteins were transferred to nitrocellulose membranes (Bio-Rad, Hercules, CA, USA) and incubated with the corresponding primary antibody (1:1000) at 4 °C overnight. Actin, Bcl-2, Bim, Bax, cytochrome c, COX4, p21, cyclin D1, cyclin E, Cdk2, Cdk4, p27, p16, and MMP-1 were purchased from Santa Cruz Biotechnology (Santa Cruz, CA, USA); Mcl-1, phospho-H2A.X (Ser139), phospho-p53 (Ser15), phospho-c-Jun, c-Jun, c-Fos, JNK, and phospho-JNK were purchased from Cell Signaling Technology (Danvers, MA, USA); MMP-9 was purchased from Abcam (Cambridge, UK); and p53 and MMP-2 were purchased from Thermo Fisher Scientific (Waltham, MA, USA). Membranes were then incubated with the relevant secondary antibodies from Thermo Fisher Scientific. Protein bands were detected using the Amersham ECL Western blotting Detection Reagent (GE Healthcare Life Sciences, Buckinghamshire, UK).

### 4.6. Cell Cycle Analysis

Cells (1.5 × 10^5^ cells/mL) were treated with hesperidin (50 µM) or SP600125 (5 µM, Calbiochem, San Diego, CA, USA), incubated for 30 min, and treated with PM_2.5_ (50 μg/mL) for 48 h. The harvested cells were washed with phosphate-buffered saline (PBS) by centrifugation at 1000 rpm for 10 min. Cells were fixed for 30 min at 4 °C in 1 mL of 70% ethanol by vortexing and then washed twice with PBS + 2 mM ethylene diamine tetra acetic acid (EDTA) by centrifugation at 1000 rpm for 10 min. Cells were stained with propidium iodide (PI) solution. For the PI solution, 100 μg/mL of PI (Sigma-Aldrich), 100 μg/mL of RNase (Biosesang, Seongnam-si, Korea), and PBS + 2 mM EDTA were used. Cells were then incubated for 30 min at 37 °C in the dark. The cell cycle phase distribution was assessed using a flow cytometer (Becton Dickinson, Franklin Lakes, NJ, USA).

### 4.7. Colony Formation

To assess colony formation, cells were seeded in a 60-mm dish at a density of approximately 500 cells/dish and treated for 7 days with hesperidin (50 µM) or with SP600125 (5 µM) and PM_2.5_ (50 μg/mL) without medium change. The medium was discarded, and the resultant colonies were stained using the Diff-Quik solution kit (Sysmex, Kobe, Japan), according to the manufacturer’s instructions. Colonies containing 50 or more cells were considered viable. Colonies were imaged and quantified using ImageJ (version 1.47; National Institutes of Health, Bethesda, MD, USA).

### 4.8. Detection of Senescence-Associated β-Galactosidase (SA-βGal) Activity

Cells were seeded at a density of 1.5 × 10^5^ cells/mL in chamber slides, incubated at 37 °C for 16 h, treated with hesperidin (50 µM) or with SP600125 (5 µM) for 30 min, and then treated with PM_2.5_ (50 μg/mL) for 48 h. Cells were then stained with SPiDER-βGal staining (Dojindo Molecular Technologies, Inc., Rockville, MD, USA) for 30 min at 37 °C and mounted on microscope slides using a mounting medium. Images were obtained using a confocal microscope (Carl Zeiss, Oberkochen, Germany). In addition, SA-βGal activity was determined using a flow cytometer (Becton Dickinson, Franklin Lakes, NJ, USA).

### 4.9. Statistical Analysis

All analyses were performed in triplicate and numerically expressed as mean ± standard error. The collected data were subjected to analysis of variance and mean separation (one-way ANOVA and Tukey’s test, respectively) using SigmaStat version 3.5 software (Systat Software Inc., San Jose, CA, USA). Statistical significance was set at *p* < 0.05.

## Data Availability

Not applicable.

## Sample Availability

Samples of the compounds are not available from the authors.
